# Sex-Specific Difference in Dynamic Balance Following Total Hip Replacement

**DOI:** 10.1093/geroni/igab019

**Published:** 2021-06-12

**Authors:** Robin M Queen, Daniel Schmitt

**Affiliations:** 1 Kevin P. Granata Biomechanics Lab, Department of Biomedical Engineering and Mechanics, Virginia Tech, Blacksburg, USA; 2 Department of Orthopaedic Surgery, Virginia Tech Carilion School of Medicine, Roanoke, USA; 3 Department of Evolutionary Anthropology, Duke University, Durham, North Carolina, USA

**Keywords:** Biomechanics, Clinical outcomes, Fall risk, Physical performance

## Abstract

**Background and Objectives:**

Total hip arthroplasty (THA) is a common surgical procedure in older adults (65 years or older). THA has high patient satisfaction, but little is known about balance and mobility limitations after surgery and if outcomes are sex-specific. This study was aimed to evaluate post-THA asymmetry during unilateral standing and a dynamic balance and reach test and test the hypotheses that balance performance would be decreased on the surgical limb and that balance deficits would be greater in women than in men.

**Research Design and Methods:**

Primary, unilateral THA (70 male, 57 female) patients completed a bilateral 10-s single-leg stance test. Sixty male but only 34 female participants could maintain unilateral balance for 10 s or greater. The cohort who successfully completed the 10-s single-limb stance test then completed a Lower Quarter Y-Balance Test in which the maximum anterior (ANT), posteromedial (PM), and posterolateral reach distances were obtained bilaterally and used to calculate the asymmetry score. All variables were compared using a mixed-model repeated-measures analysis of variance (sex by limb), while independent samples *t* tests were used to assess sex-specific asymmetry.

**Results:**

Women failed single-leg stance at a higher rate than men (85.7% vs 59.6%; *p =* .001). Reach distance was different between limbs for all reach directions (*p* < .004) with greater reach distance on the nonoperative limb for all patients. Men had a greater reach distance in the ANT (*p =* .004) and PM (*p =* .006) directions.

**Discussion and Implications:**

These results indicate that post-THA, the operative limb and female patients have greater balance limitations. These results are novel and reveal sex-specific patterns that emphasize the need for sex-specific postoperative rehabilitation programs to improve long-term outcomes, especially in older adults with muscle weakness and balance deficits.

**Translational Significance:** This study reveals significant deficits in balance after hip replacement that are higher for older adult women than older adult men, putting older adult women at a higher risk for falls and injury after surgery. Clinical and geriatric care professionals can use these data to formally assess risk through rigorous testing protocols involving single-leg balance and Y-Balance Tests and design strengthening and fall prevention protocols for any of their patients older than 65 years following THA.

## Background and Objectives

Total hip replacements are currently the preferred treatment for painful or debilitating hip arthritis and are often described as successful based on patient satisfaction and implant survival (Arden et al., 2011), two-thirds of which are performed on patients older than 65 years (Crawford & Murray, 1997), and represent an important factor in geriatric care and long-term outcomes for older adults. However, there are few data on how successful these surgeries are in restoring balance and mobility to relatively healthy levels and if this success differs for men and women. This is a focus of this paper and an important area in this field (Leopold et al., 2014).

Women have been reported to have higher levels of hip osteoarthritis, have higher rates of total hip arthroplasty (THA) surgery (Edwards et al., 2013; Johansson et al., 2016), and be at a greater fall risk than men (Gale et al., 2016; Stevens & Sogolow, 2005; Wei & Hester, 2014). Gale and colleagues (2016) observe that one, of many, risk factors for falls is the failure to perform a one-legged balance test. In addition, studies on the need for walking aids and pain medication following THA suggest that women have a higher rate of moderate-to-severe activity limitations up to 5 years following surgery when compared to men (Peterson et al., 2017; Singh & Lewallen, 2009).

Although THA increases range of motion and to a lesser extent spatiotemporal parameters compared to preintervention status (Miki et al., 2004; Queen, Butler, et al., 2011), the surgical hip generally is restricted relative to the nonsurgical hip and the majority of patients show asymmetric loading patterns that are associated with deficits in balance and proprioception that persist for at least 2 years following surgery (Bennett et al., 2008; McCrory et al., 2001; Queen et al., 2015; Queen, Watters, et al., 2011). Few studies have examined sex-specific differences in gait following THA and those studies did find significant though limited differences between men and women before and after surgery (Brunner & Foucher, 2018; Foucher, 2016; Peng et al., 2021).

One area that needs attention is whether men and women achieve the same levels of balance and mobility after surgery? Although women self-report more pain and disability prior to THA, there are no differences in self-report compared to men 12 months after surgery (Mannion et al., 2015). Although a 10-s single-limb balance test is used as a measure of potential success in balance and range of motion, it is not known whether those persons who are able to perform such a test after surgery still have balance limitations and if those limitations differ by sex.

The purpose of this study is to determine if sex-specific differences exist in static and dynamic balance following THA. Based on previous studies (Gale et al., 2016; Queen et al., 2015; Queen, Butler, et al., 2011), we designed a two-stage study that begin with a simple 10-s single-limb balance test and was followed with a more comprehensive balance test that explores mobility and balance.

We hypothesized that women would (a) have significantly worse unilateral static balance and (b) have a more limited dynamic balance, as measured, respectively, by a single-leg stance test and by the Lower Quarter Y-Balance Test (YBT-LQ) following THA when compared to men at equivalent postsurgery time points. The YBT-LQ is a dynamic test that reflects reductions in mobility and balance associated with musculoskeletal injury, pathology, and surgical intervention (Gribble et al., 2012; Plisky et al., 2006).

## Research Design and Methods

### Study Design

We identified 127 patients who underwent primary, unilateral total hip replacement (70 male and 57 female) from a prospective quality improvement database of total joint arthroplasty patients (Level of Evidence 3). Institutional Review Board approval was obtained to use the database to identify a population of patients who had undergone a THA at least 12 months prior to testing, had no diagnosed or symptomatic osteoarthritis at any other lower extremity joint, and were able to ambulate without the use of an assistive device. Patients were excluded from the data set if they had contralateral lower extremity pain, contralateral lower extremity joint degeneration, prior lower extremity joint replacement or spinal surgery, or a history of neurologic or vascular disorders that affected balance, gait, or activities of daily living. All data were obtained as part of the patient’s standard clinical assessment at a scheduled clinic visit between 12 and 36 months postoperatively.

Two separate tests were performed for this study. The first was a single-leg balance test to see if the participant could stand on one leg for longer than 10 s. Values were treated as binary with success being greater than 10 s and failure being less than 10 s.

The second phase involved a dynamic YBT-LQ test that was conducted on participants who passed the single-leg balance test. The YBT-LQ is derived from the Star Excursion Balance Test and employs three reach directions: anterior (ANT), posteromedial (PM), and posterolateral (PL; Plisky et al., 2009) and results from this measure have been correlated with lower extremity musculoskeletal injury (Gribble et al., 2012; Plisky et al., 2006). In addition, previous research has suggested that a lower overall score or a greater amount of reach asymmetry are both associated with an increased risk of injury in certain athletic populations (Herrington et al., 2009; Plisky et al., 2006; Smith et al., 2015). While a number of studies on the YBT-LQ have been conducted in young, athletic populations (Chimera et al., 2015; Coughlan et al., 2012; Engquist et al., 2015; Plisky et al., 2006), only a few have assessed dynamic balance in healthy older adult populations (Lee et al., 2014, 2015), and no previous studies have used the YBT-LQ to examine dynamic balance in postoperative THA patients.

All assessments were completed by test administrators who had been trained in completing the YBT-LQ. All testing was completed with participants barefoot according to previously published YBT-LQ procedures (Shaffer et al., 2013). First, each participant was asked to complete a 10-s single-leg stance test on each limb. Only those patients who could pass this test were allowed to proceed with YBT-LQ testing.

**Figure 1.**
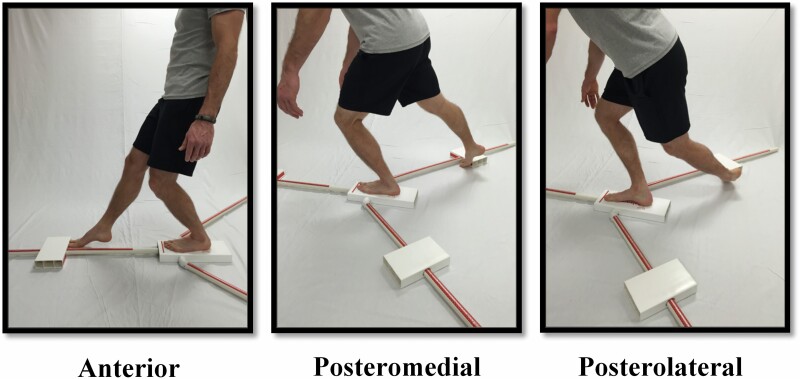
Lower extremity Y-Balance Test.

Before the YBT-LQ was completed, the test administrator reviewed the instructions for the completion of the YBT-LQ. During the YBT-LQ, the participant was instructed that they had to remain in unilateral stance on the stance platform while pushing the reach indicator in three independent directions (ANT, PM, and PL) (Figure 1). Each participant began the YBT-LQ by standing with the one foot (stance foot) on the stance platform with the long axis of the foot in line with the ANT reach pole and the foot stationary. In order to standardize the protocol for all participants, the right limb was used as the stance limb for the first set of trials. Each participant completed three reach trials in the ANT direction while standing on the right foot. Then the participant was asked to stand on their left foot and complete three reaches, this time pushing the reach indicator with the right foot. The same right and the left limb pattern was then completed for three reach trials in the PM and finally the PL directions. After completing the three practice trials in each reach direction on each foot, the participants were allowed up to a 5-min rest before being asked to complete the same procedure again with three reaches on each foot in each reach direction.

In order for the reach to be considered successful, the following criteria had to be achieved: (a) the participant was able to push the reach indicator, using the most distal portion of the foot, as far as possible while maintaining balance and returning back to the starting position, without touching the reach foot to the ground; (b) a kick was not used to make the reach indicator move; and (c) the foot was not placed on top of the reach indicator while pushing the indicator or when trying to return to the starting position (Plisky et al., 2009; Shaffer et al., 2013). A trial was repeated if any of these criteria were not met. The rest period between each reach trial was approximately 30 s, enough time for the administrator to record the data and return the reach indicator back to the starting position. Limb length was measured with the participant in supine position from the inferior aspect of the ANT superior iliac spine to the inferior aspect of the medial malleolus, in order to normalize for anatomic differences in limb length (Normalized Reach Distance = Reach Distance/Limb Length).

### Outcome Measures and Statistical Approach

The primary variables of interest for this study were (a) pass or failure of the single-leg stance test, (b) the mean normalized reach distance for both the operative and nonoperative limbs in each of the three reach directions, (c) reach asymmetry, and (d) the YBT-LQ composite score. Reach asymmetry was defined as the difference between the operative and nonoperative limbs in each of the reach directions. The composite score was calculated as the sum of the three maximum reach directions divided by three times the limb length. Sex- and limb-specific differences in dynamic balance were assessed by a series of mixed-model repeated-measures analyses of variance. Chi-squared analysis was used to determine sex differences in the completion of the 10-s single-leg stance screening test and an independent samples *t* test was used to compare the operative and nonoperative difference score between men and women. All statistical analyses were performed using JMP, version Pro 10.0.0 (SAS Institute Inc., Cary, NC) with the level for statistical significance set at (α *=* .05).

## Results

### Demographics and Results of Single-Leg Balance Test

No significant differences existed between the sexes for age, body mass index (BMI), or time since surgery for the entire patient cohort (Table 1). When examining the sex-specific differences in static balance, of the original 70 men and 57 women, men were able to pass the 10-s single-leg stance screen significantly more than the women (85.7% vs 59.6%; *p* = .001).

**Table 1.** Subject Demographics and Sex-Specific Results of Single-Leg Balance Test

				Female			Male			Male-to-female comparison	
Variables	Female (*n* = 57)	Male (*n* = 70)	*p*	Fail (*n* = 23)	Pass (*n* = 34)	*p*	Fail (*n* = 10)	Pass (*n* = 60)	*p*	Fail *p*	Pass *p*
Age (years)	57.7 (15.3)	52.7 (15.1)	.067	65.5 (11.4)	52.35 (15.4)	<.001*	66.2 (8.0)	50.4 (14.8)	<.001*	.856	.556
Body mass index	26.7 (6.2)	29.7 (5.6)	.053	30.9 (8.4)	27.5 (6.1)	.108	32.0 (6.7)	29.5 (5.2)	.142	.700	.119
Months postoperation	30.0 (16.2)	21.7 (16.7)	.071	20.2 (16.0)	36.7 (30.4)	.051	15.5 (6.9)	22.8 (17.6)	.027*	.424	.042*

*Note*s: Subject demographics for the entire cohort (regardless of screen test results), female subjects who passed or failed the screening test, and male subjects who passed or failed the screening test. Mean and 1 *SD* (in parentheses) are presented. *p* Values compared by gender and then by screen test for female and male subjects are provided in the furthest right column. In addition, *p* values are given for a comparison of the values between genders for subjects that failed the screen test and subjects that passed the screen test.

When comparing those participants who passed the 10-s single-limb balance test, men and women who failed the test were significantly older than those that passed by on average 14 years (*p* < .0001 in both cases). There were no significant differences in age or BMI, or time since surgery when comparing men and women who passed the balance test or men and women who failed the balance test.

There were differences in months since surgery when comparing men and women who passed the balance test. Men who passed the balance test were on average 16 months longer past surgery than men who failed (*p =* .027). There was a similar gap for women, but due to high variability, this was not significant (*p =* .052). There was also a significant difference when comparing people who passed by gender, with women who passed the balance test being on average 15 months further out from surgery than men who passed (*p =* .042). This finding suggests that women had longer to heal and gain strength. It is worth noting again in this context that there was no difference in age between men and women who passed the balance test (*p =* .558).

### Results of Y-Balance Test

Based on the successful completion of the 10-s single-limb balance test, 94 patients (60 male and 34 female) were eligible to complete the YBT-LQ. There were an additional 20 male and nine female participants who were not able to complete all reach directions of the YBT-LQ and therefore the final statistical analysis for the YBT-LQ comparison was completed with 65 patients (40 male and 25 female).

**Figure 2.**
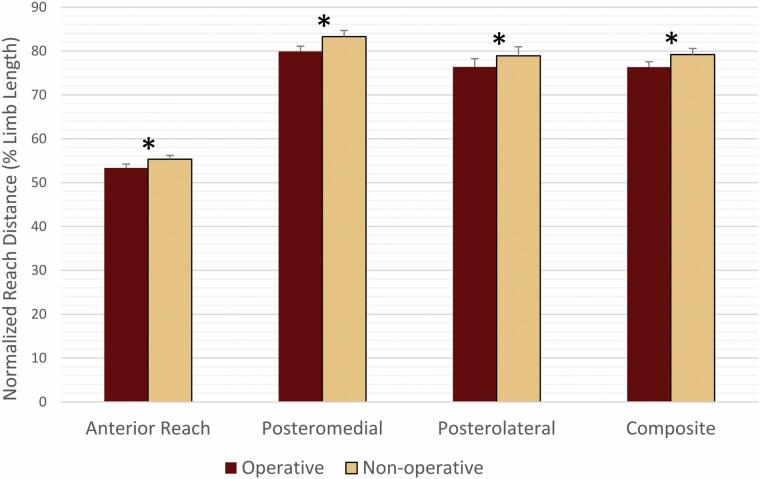
Reach asymmetries following total hip replacement between the operative and the nonoperative limb for each of the three reach directions and the composite score. *p < .004.

No significant sex-by-limb interactions existed for any of the variables of interest. A difference in normalized reach distance existed between the operative and nonoperative sides for each of the reach directions (ANT: *p =* .001, PM: *p* < .001, PL: *p =* .004) as well as the composite score (*p* < .001) with the nonoperative limb having a greater reach distance (Figure 2).

**Figure 3.**
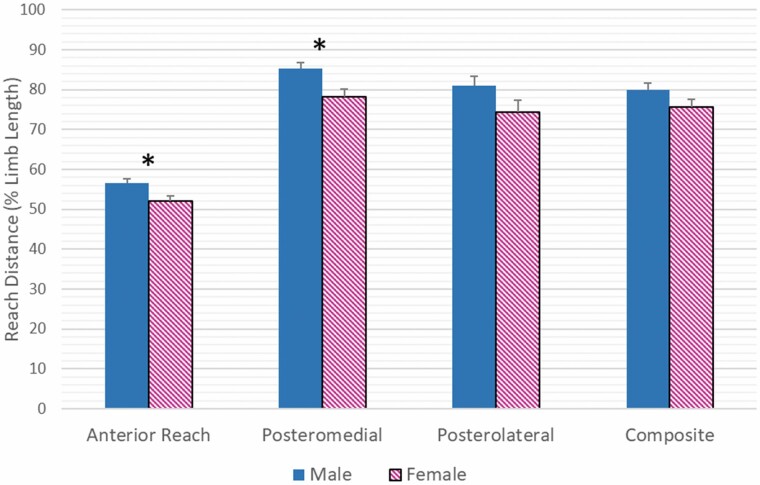
Sex-specific differences in reach asymmetries following total hip replacement for each of the three reach directions and the composite score. *p < .006.

When comparing males and females (main effect for sex), the males had a greater normalized reach distance in the ANT (*p =* .009) and PM (*p =* .006). The difference between the sexes in the PL (*p =* .097) and the composite score (*p =* .088) was small (5%) and statistically not significant (Figure 3).

## Discussion and Implications

THA is a common orthopedic procedure that has high patient satisfaction (Arden et al., 2011), but little is known about the degree to which hip range of motion and full balance are restored and whether success in these outcome measures differ between men and women. This study tested two hypotheses related to sex-specific differences in balance following THA. The first was that older adult women would fail a 10-s single-limb balance tests at a higher rate than older adult men. The second was that women would demonstrate a decrease in dynamic balance (limited reach distances) on a YBT-LQ.

The first sex-specific hypothesis was supported unequivocally. In the current study, only 74% of patients were able to pass the 10-s single-leg stance screening assessment, which is only slightly higher than the 63% that has been previously reported (Butler et al., 2015). In contrast, men were able to pass this test at a significantly higher rate than women (85.7% vs 59.6%; *p =* .001). Although the original sample contained more women than men, which reflects the patient population accurately, more men than women were able to move on to the second phase of testing. It is also the case that the men and women who moved to the second phase were the same age, but the women were more months past surgery than the men in the study.

The second part of the study demonstrated that following surgery, female patients have greater balance deficit when compared to men during the dynamic balance assessment (YBT-LQ). Balance deficits exist at a significantly higher level in women compared to men for reach distance normalized to limb length in the ANT and PM directions. These results could indicate weakness in hip and quadriceps muscles in older adult women, which are essential for completing the reaching task.

The overall results of this study show that even what are considered high-functioning THA patients (those that were able to complete a 10-s single-leg stance test bilaterally) are more limited in dynamic balance on the operative limb when compared with the nonoperative limb independent of sex (*p* < .004). Previous research has indicated that following THA, patients demonstrate an asymmetrical movement and loading pattern during both level walking and stair climbing (Lugade et al., 2010; McCrory et al., 2001; Queen et al., 2015; Queen, Butler, et al., 2011; Queen, Watters, et al., 2011; Talis et al., 2008). These limb balance asymmetries appear to be associated with previously reported gait disabilities and asymmetric patterns of loading and walking (Lugade et al., 2010; McCrory et al., 2001; Queen et al., 2015; Queen, Butler, et al., 2011; Queen, Watters, et al., 2011; Talis et al., 2008). Previous literature has reported that balance and stability are restored following THA (Brauner et al., 2014; Nallegowda et al., 2003), but these assessments have been limited to bilateral balance assessments which allow for the use of the nonoperative limb to compensate for any weakness or instability on the operative limb. When examining unilateral static balance, it is clear from this study as well as previous studies (Butler et al., 2015) that THA patients have limitations in single limb stance following surgery.

These results show clear patterns but there are some important limitations to this study including the relatively small sample size and the use of prospective clinical data. With regard to the latter, we do not know about balance deficits prior to surgery, though we do know that men and women were well age- and size-matched for both parts of the study. In addition, this study could not control for the effect of surgical approach, which can impact both postoperative complications and postoperative walking mechanics (Jolles & Bogoch, 2006; Queen et al., 2013, 2014; Ritter et al., 2001). Unfortunately, the sample here was not diverse enough in surgical approach to assess the impact of surgical approach on dynamic balance.

The differences in balance between males and females following THA are stark. They indicate the need for sex-specific postoperative rehabilitation programs that focus on patient-specific balance deficits in order to specifically target areas of weakness in balance, proprioception, and strength. It is already well recognized that older adult women are at a greater risk for falls (Gale et al., 2016, 2018). The current study adds to that by recognizing that although THA is a critical and successful treatment for osteoarthritis that improves patient life quality, it also decreases balance and puts older adult patients at a higher risk for falls. This risk is higher for older adult women compared to older adult men.

The implication of these data are differences in muscle strength, which should be assessed as part of a standard of care. There remains debate about the degree to which muscle strength and balance are related, and this area is both exciting and expansive. Fukagawa and colleagues (1995) made a strong assertion that muscle strength was a key factor in balance and falls. This remains a consensus view (de Labra et al., 2015; Papalia et al., 2020; Šarabon & Kozinc, 2020), though it should be noted, for example, that the relationship between strength and Y-Balance Tests are not absolute. Some studies have found a weak correlation between hip strength and Y-Balance results (Lee et al., 2014, 2015).

Nonetheless, there is reason to argue for strength training as an opportunity to improve balance. The nature of training remains a debate. Moreland and colleagues (2004) confirm that exercise broadly improves strength and balance, but argue that it is unclear what kind of exercises are most effective. Both resistance training and training with complex balance challenges have been advocated as effective for improving strength and balance (Šarabon & Kozinc, 2020; Sherrington et al., 2008).

It is beyond the scope of this paper to address this complex issue, but some recommendations can be made as part of a translational perspective. The balance abilities of older adults who have undergone surgery can be effectively and conveniently assessed and health professionals should be encouraged to do so for extended periods after surgery and engage in fall prevention strategies for those patients that show deficits. The use of single-leg stance tests as a first assessment of balance and risk and then YBT-LQ as a secondary assessment are valuable clinical tools to examine the impact of surgery on physical function without the need for three-dimensional motion analysis.

In association with these assessments, clinical professionals can recommend and supervise strength training exercises, especially those using resistance training which has been shown to improve single-leg balance and Y-Balance results (Šarabon & Kozinc, 2020), as part of a plan to help restore balance symmetry and improve outcomes. Particular attention should be given to female patients in any age group.

We propose that the THA population should be assessed at 6 weeks postoperation if the patient is able to maintain adequate balance. If there is not adequate balance at that point, then an exercise progression focused on balance should be initiated. The participant would be reassessed every few weeks and exercises would be adapted based on progression with a focus on improving overall balance and restoring side-to-side symmetry.
